# The role of organizational attractiveness in an internal market-oriented culture (IMOC): a study of hospital frontline employees

**DOI:** 10.1186/s12913-019-4144-8

**Published:** 2019-05-14

**Authors:** Terje Slåtten, Gudbrand Lien, Peer Jacob Svenkerud

**Affiliations:** Inland Norway University of Applied Sciences Campus Lillehammer, 2604 Lillehammer, Norway

**Keywords:** Organizational attractiveness, Employee engagement, Turnover intentions, Service quality provision, Internal-market-oriented culture (IMOC), Frontline employees, Hospitals

## Abstract

**Background:**

Hospitals need to understand how to reduce their frontline employees’ turnover rate as well as how to positively engage them and improve their service. Central to these issues, we find, is the employees’ perception of their organization’s attractiveness. This objective of this paper is to clarify how the role of organizational attractiveness relates to frontline employees’ perception of their internal market-oriented culture as well as their turnover rate, engagement, and service quality. To our knowledge, no previous research has explored the role of organizational attractiveness from a frontline employee perspective in health-service organizations.

**Methods:**

The conceptual framework we developed was tested in a quantitative study. We sent a questionnaire to nurses in several public hospitals in Norway. We then analyzed the data with confirmatory factor analysis and structural equation modeling in Stata. Further, we performed multi-group comparisons to test heterogeneity in personal characteristics. The indirect effects were tested by mediator analyses.

**Results:**

**We made three main findings.** First, organizational attractiveness has a significant positive effect on frontline employees’ engagement (β = 0.833) as well as on the service quality they provide to hospital patients (β = 0.472). Additionally, it significantly lowers their turnover rate (β = − 0.729). Second, the ‘internal market-oriented culture’ (IMOC) has a significantly positive effect on organizational attractiveness (β = 0.587) and explains a total of 35% of the variance in organizational attractiveness. Third, organizational attractiveness fully mediates the relationship between “internal market-oriented culture” (IMOC) and frontline employees’ engagement and the service quality they provide to patients, and it partially mediates the relationship with the turnover rate.

**Conclusions:**

This study proves that organizational attractiveness is vital for hospital managers to focus on, as it affects employees’ perception of whether the organizations is a great place to work. It reveals the need for those same managers to develop an internal market-oriented culture (IMOC) directed toward hospital frontline employees, as it has both a direct effect on organizational attractiveness and an indirect effect on employees’ engagement, turnover intention, and service quality.

**Electronic supplementary material:**

The online version of this article (10.1186/s12913-019-4144-8) contains supplementary material, which is available to authorized users.

## Background

“Organizations are collections of people joined together in pursuit of a common cause and it is people who create value” ([1], p. 42). In healthcare organizations, a critical part of the human resource base are those employees working on the frontline. Landry et al. note that “The delivery of health care services relies on an appropriate and sustainable health human resource base” ([2], p. 1). Because they are working “face to face” with patients, hospitals should especially prioritize focus on them. *Fortune*, which annually identifies the 100 best companies to work for, states: “employees who say they have a great place to work were four times more likely to say they’re willing to give extra to get the job done” [3]. This being so, healthcare organizations (e.g., hospitals) would be wise to identify the specific factors associated with their employees’ perception of organizational attractiveness (OA), especially those working on the frontline.

Worldwide, healthcare organizations such as hospitals face numerous challenges [4] that are either directly or indirectly related to their employees’ perception of organizational attractiveness (OA). One such challenge is the sheer increase in the number of hospitals. In Norway, for example, private hospitals have multiplied in recent years, leaving job-seekers with many more alternatives to choose from. Furthermore, that same spike in hospitals also leads to increased competition in recruiting new people.. For some less attractive hospitals, it means facing a higher turnover rate among their employees, especially their frontline ones, such as nurses. In OECD countries, health organizations report high levels of turnover among nurses [5], which they describe as an “ongoing problem” ([5], p. 1180). Research has shown that unstable staffing has negative consequences, including lower resident satisfaction [6] and a decrease in the quality of care [7]. Moreover, the level of job satisfaction is found to be linked to employee turnover intentions [8]. The same is true of service employees’ perception of the service quality offered to customers [9]. All of which supports the importance of focusing on frontline employees’ perception of organizational attractiveness (OA) in their place of work—in our case, hospitals.

The area of research that studies OA is “employer branding.” As defined by Berthon et al., it is the “sum of a company’s effort to communicate to existing and prospective staff that it is a desirable place to work” ([10], p. 153). Employer branding is all about increasing OA. Its objective is to differentiate one’s own organization from competing organizations as the more satisfying place to work.

The study of OA, within the domain of employer branding, has rightly become an emerging area of research. But much of it has limited its focus to how best to attract potential job applicants (e.g. [11, 12]). This narrow focus has led to a neglect of studying OA from a current employee’s perspective. This underlines the importance to understand what makes an organization attractive to its current employees, especially those on the frontline.

To our knowledge, there has been no previous study in healthcare research, with but one exception that has focused on OA from a current employee perspective and with a specific focus on frontline employees. That one exception is the study of Trybou et al., [4]. Although their study identified several interesting factors associated with OA, it did not examine how frontline employees’ perception of organizational culture is associated with OA. Moreover, those researchers did not examine any effects of OA; they only proposed examples of potential effects or outcomes that “pose interesting possibilities for future research” (Trybou et al., [4] p. 8). There’s clearly a need for more research into several aspects related to OA from a frontline-employee perspective.

Our study has three objectives. First, and most broadly, we aim to contribute to an emerging field within health-service research that focuses on OA. Second, and more specifically, we aim to show how frontline employees’ perception of OA is linked to different types of important job-related outcomes. This will in turn show hospital managers the value of focusing on OA as a key part of their overall employer branding program. Third, we aim to show how a frontline-focused culture in organizations—that is, an internal market-oriented culture (IMOC)—is linked to employees’ perception of OA. This will also reveal whether the linkage between IMOC and different types of job-related outcomes is mediated through OA. No previous study within health-service research has focused on these aspects.

We will begin by presenting the conceptual model of our study. We will then describe and define OA as well as the other constructs that are either directly or indirectly linked to OA. Then we will lay out the methodology and findings from our empirical study. We will conclude with a discussion of our findings and offer several suggestions for future research.

## Conceptual model

**Fig. 1:**
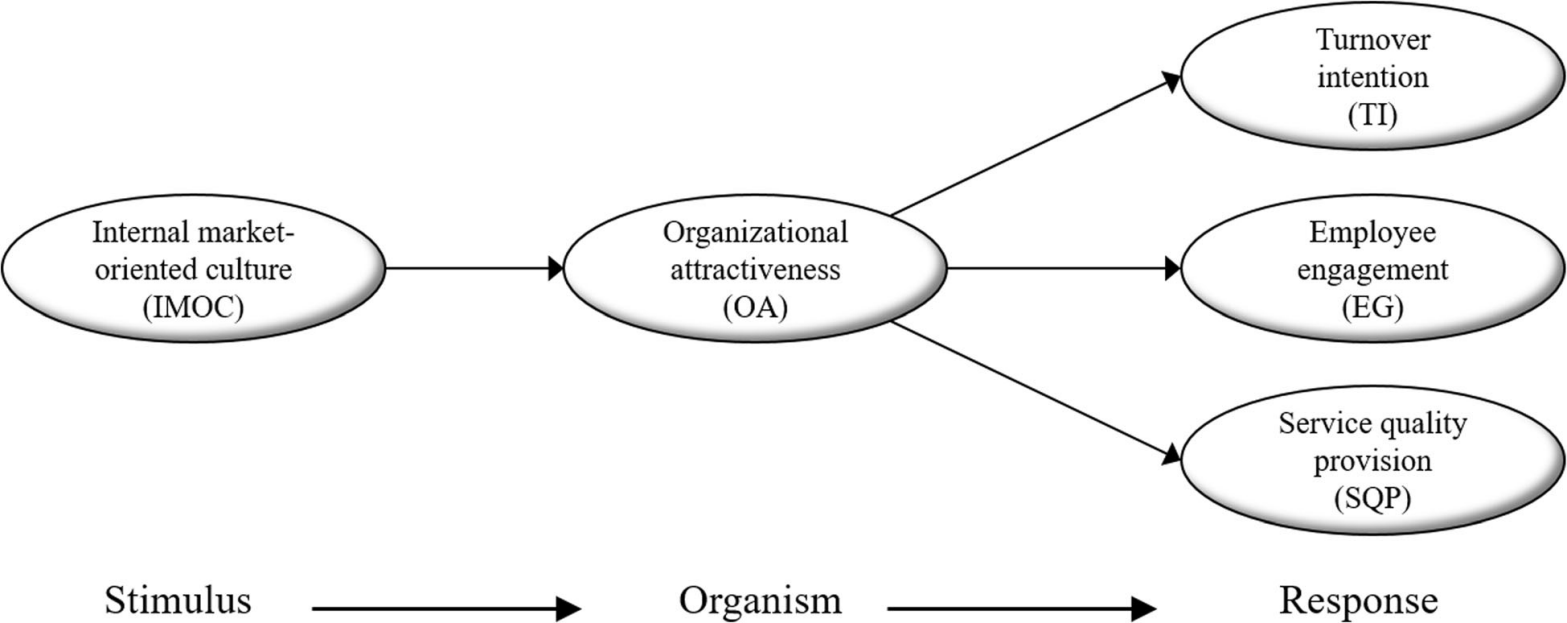
Conceptual model to analyze frontline employees’ perception of organizational attractiveness.

Figure 1 illustrates our conceptual model, which is inspired by the logic of the elements that constitute the generic *Stimulus–Organism–Response* (SOR) model [13]. That SOR model helps one visualize how environmental factors (S/*Stimulus*) affect a person’s perception or attitude (O/*Organism*), which in turn cause different effects (R/*Response*) related to the person. As seen at the bottom of Fig. 1, the SOR elements are connected in a specific and directional cause-and-effect manner.

Following the line of reasoning shown in Fig. 1, our aim is to explore both the direct and indirect effects of OA from the perspective of an organization’s existing frontline employees. Specifically, the *Organism* element (O) in Fig. 1 is represented by the concept of OA. The *Stimulus* element (S) is represented by an organization’s IMOC. The *Response* element (R), meanwhile, is represented by three factors—turnover intentions (TI), employee engagement (EG), and service-quality provision (SQP).

In the following sections, we discuss each of the SOR elements and hypothesize linkages between the different concepts.

### Organizational attractiveness (OA)

In this study, OA represents the *Organism* element. In today’s competitive healthcare environment, organizations should “strive to be attractive employers” ([14], p. 474)—that is to say, an attractive organization in the eyes of both current and potential new employees. In this study, we focus on OA from an internal perspective (the current employees’) and consequently exclude an external focus (attracting new applicants). Generally, whenever a person considers something as potentially attractive, their initial overall evaluation then gets subjected to a comparison or contrast with other relevant and very specific features. To be considered truly attractive, the thing must be either (a) better than the second-best relevant and important alternative or (b) at least be within a person’s individual zone of tolerance of what one finds acceptable or attractive. The *Oxford English Dictionary* defines “attractiveness” as “the possession of qualities or features that arouse interest.” So for an organization to be considered attractive, it should manifest enough desirable qualities to make it rate as a great prospect.

The literature offers us diverse definitions of organizational or employer attractiveness. But what they all have in common are the advantages and satisfactions one finds in working for a company [15, 16]. Take, for example, the definition of Berthon et al., who define organizational or employer attractiveness as “the envisioned benefits that a potential employee sees in working for a specific organization” ([15], p. 156). Ambler and Barrow, meanwhile, suggest three potential dimensions of benefit in working for a company: (1) psychological, (2) functional, and (3) economic [17]. Berthon et al., going even further, propose five dimensions of organizational or employer attractiveness: (1) interest value, (2) social value, (3) economic value, (4) development value, and (5) application value (see [15] for more information about the content of each dimension). While most researchers operationalize attractiveness in terms of benefit or value, others do so in terms of instrumental and symbolic attributes. For example, in Lievens and Highhouse [18], instrumental attributes refer to tangible or relatively objective aspects of what an organization actually offers to potential applicants (e.g., salary, flexibility, location), while symbolic attributes refer to subjective and intangible aspects offered to potential applicants (e.g., prestige, organizational culture, innovation degree). Although there are obviously various different ways to describe organizational or employer attractiveness, most descriptions share much the same focus and aim and can thus be considered near-synonyms.

Clearly, the presumed work benefits offered by a particular company shed light on the concept of organizational attractiveness in general. But from an internal perspective (i.e., that of a company’s current employees), those benefits might only be superficial gauges of someone’s perception of organizational attractiveness rather than solid proof that they indeed perceive that organization as a lovely place to work. Following this line of reasoning, the different aspects or dimensions of benefits, such as application value and social value, can be seen as (only) trigger factors that are causes of organizational attractiveness, and do not necessarily confirm the concept of organizational attractiveness from the perspective of current employees. Previous research has largely been dominated by studying organizational attractiveness using the Employer Attractiveness Scale of Berthon et al. [14]. Furthermore, most studies have adopted an external perspective when investigating organizational attractiveness, with a specific focus on understanding the attributes of an organization that are considered attractive for *potential* candidates. As noted, studying organizational attractiveness from an *internal* and *current employee* perspective has largely been neglected, the one real exception being Trybou et al. [4], whose work has motivated and guided us in developing the concept of organizational attractiveness in this present study. Trybou et al. aimed to identify determinants of attractiveness for people already working in an organization. As in our work, they used frontline hospital employees as the empirical context. To their credit, they [4] carefully distinguished between the conceptualization of hospital employees’ perception of organizational attractiveness and the triggering factors or attributes that cause or “drive” employees’ perception of organizational attractiveness. Interestingly, both the overall categories of attributes of organizational attractiveness (e.g., economic, relational, professional attributes) and the subcategories of specific attributes (e.g., pay benefits, financial benefits, prestige) that were used as drivers of organizational attractiveness in Trybou et al. [4] nicely match how most existing studies have conceptualized organizational attractiveness. Consequently, this supports our argument that there is good reason to question the conceptualization of organizational attractiveness in previous research, especially when the aim is to study organizational attractiveness from an internal and current employee perspective. Thus, the concept of organizational attractiveness concerns a person’s evaluation of how attractive they perceive an organization to be. Therefore, in our conceptualization, we follow Trybou et al. [4] and define organizational attractiveness as an *attitudinal* construct. Specifically, organizational attractiveness can be defined as “employees” attitude toward (1) choosing the same organization or employer again if presented the choice, and (2) recommending the organization or employer to someone you know well. It is reasonable to assume that these two aspects capture well a core objective for any company to strive toward. Consequently, by studying organizational attractiveness as an attitude, one may capture both the direction of the attitude (positive or negative) as well as the strength of the attitude of current employees in the organization. Our chosen definition corresponds well with how Aiman-Smith et al. frame and define “attractiveness.” They call it “an attitude or expressed general positive affect toward an organization, toward viewing the organization as a desirable entity with which to initiate some relationship” [19]. Moreover, defining organizational attractiveness as an attitude also matches how the *Organism* element is described based on the generic SOR model (in Fig. 1), which defines attitude as “internal intervening processes and structures [that] consist of perceptual …. feeling and thinking activities” [20].

### Direct effects of OA

In Fig. 1, OA is linked to three direct effects or responses: (1) turnover intentions (TI); (2) employee engagement (EG); and (3) service quality provision (SQP). We will now look at each of these direct effects in turn.

#### Turnover intentions (TI)

In Fig. 1, TI are suggested as a direct effect of OA. We have two reasons for including turnover intentions in this study. First, as noted, some healthcare employees, like nurses, experience high turnover [5]. Second, research finds that TI is a strong predictor for the actual (behavioral) manifestation of employee turnover in organizations across industries [21]. Consequently, TI are highly relevant as a part of the conceptual model. In this study, TI refer to employees’ psychological response to organizational conditions [22], and also to their consideration (or thoughts) about actually quitting the organization. Specifically, TI refer to “the final cognitive step in the decision making process” ([23], p. 23) and imply that employees are open to and have thoughts about leaving the organization and seeking alternative employment.

Surprisingly, no research has linked the concept of OA to employee TI. Most studies have limited their focus to recruitment [24]—and, specifically, how best to attract potential applicants to the organization [11, 12]. Consequently, the direct effect of OA on TI of those employees already working in an organization remains unexplored. But it’s reasonable to assume that OA is able to affect employees’ consideration about leaving an organization. As defined in this study, OA implies that an employee has mostly positive attitudes toward their organization, captured in the popular expression *this is a great place to work!* Based on this reasoning, OA can be considered as a potential source of positive “power that … encourages existing employees to stay” [10] with the organization. OA is thus able to greatly reduce thoughts about employee TI. Hence, in this study, we propose the following:

- **Hypothesis 1:** *Organizational attractiveness is negatively related to employee turnover intentions.*

#### Employee engagement (EG)

In Fig. 1, EG is suggested as another direct effect of OA. In our present study, EG is based on the relatively well-established definition of Schaufeli et al., who define engagement as “a positive, fulfilling, work-related state of mind” ([25], p. 74). Engagement is characterized by three things: (1) vigor, (2) dedication, and (3) absorption. Vigor describes a person’s energy and mental resilience while working. Dedication describes a person’s enthusiasm and sense of significance regarding their work. Absorption describes how deeply engrossed a person is in their work [25].

Many organizations, like hospitals, operate in a highly competitive environment. Frontline employees (especially those carrying major responsibilities) are a critical resource in such an environment. Consequently, “an organization [e.g. hospital organization] that has the ability to … bring out the best in employees [e.g., frontline employees] will have a competitive advantage over opponents” ([26], p. 34). Previous research has suggested that EG in an organization, on both the individual and collective level, essentially reflects that organization’s ability to achieve a competitive advantage [27, 28]. To engage employees in an organization, especially a monolithic corporate one, is not necessarily an easy task. Kaye and Jordan-Evans state that “the challenge today is not just retaining talented people, but fully engaging them, capturing their minds and hearts at each stage of their work lives” [29]. Consequently, it is important to elucidate the extent to which OA can be characterized as a “driver” with a direct effect on EG.

To our knowledge, no previous research has explored any linkage between OA and EG. Nevertheless, common sense lets us assume that the two concepts are related, and social-identity theory supports that assumption. A core idea in social-identity theory is that a social identity exists whenever a person (such as an employee) perceives that he or she belongs to a specific group (such as an organization). Naturally, the level of any person’s perception of identity or belonging to their group will vary in strength and intensity. This type of organizational identification is suggested to be a “special form of social identification” ([30], p. 4). When applying social-identity theory to an organizational context, it’s reasonable to again assume a varying degree of how strongly individual employees perceive their identification or ‘magnetic association’ with their company. Consequently, the variations in employee perception of organizational identification overlap or equate with the idea that employee perception of OA can also vary across individual employees in an organization. Similar to this study concept of OA, defined as a cognitive (or attitudinal) construct and thus “viewing the organization as a desirable entity” [20], organizational identification is about the “cognitive connection between the individual and the organization” ([30], p. 4). Chen et al. argue that there’s an overlap between organizational identification and OA, and note that organizational identification reflects employees’ assessment of organizational attractiveness [30]. Given this line of reasoning and social-identity theory, employees who strongly identify with their organization, and thus have a positive attitude toward its attractiveness, are likely to make their best effort to benefit that organization [30]. In the present study, this “best effort” is reflected in the concept of EG, their active engagement. So when employees view their organization as an attractive workplace, this drives EG in it in a positive manner, and is reflected in their enthusiasm, engrossment, and mental resilience while performing their work role. Thus our second hypothesis:

- **Hypothesis 2:** *Organizational attractiveness is positively related to employee engagement.*

#### Service quality provision (SQP)

In Fig. 1, SQP is suggested as a direct effect of OA. In professional service firms, such as hospitals, it’s critically important that they deliver exemplary service to their customers/patients, some of whom may be facing life-or-death issues. Frontline employees such as nurses are of course essential contributors to patients’ overall satisfaction with the facility. Indeed, nurses “tend to have the longest and closest contact with patients” ([30], p. 1). The nursing staff generally constitute 40–60% of the total human resources in a healthcare organization [30]. They are therefore a core resource for hospitals with respect to their reputation, image, and competitiveness. For this study, however, we have switched the usual perspective—from receiver to giver. SQP concerns the perception of frontline employees (nursing staff) as to the “design and delivery of knowledge-intensive solutions” ([31], p. 1603) they deliver to hospital patients—in other words, how they themselves view their nursing care. Note that we are differentiating here their own view from their patients’ separate evaluation of the service they’ve received. To avoid potential confusion, we add the word “provision” to “service quality” (thus SQP) in order to signal our intended perspective. To repeat, SQP refers to frontline employees’ own assessment as to how well they’re serving their patients.

No previous research has explored the direct effect between OA as defined here and SQP from a hospital frontline perspective. But we assume, we think reasonably, that frontline employees who have a positive attitude toward their organization’s attractiveness are more happy to devote extra time and energy to work for the best of their organization (such as delivering excellent service to patients) compared with those frontliners who have a less positive (or even negative) attitude toward the attractiveness of their workplace. Moreover, based on social-identity theory, when frontline employees identify positively with their organization, in addition to EG, it drives frontline employee SQP to hospital patients. Finally, previous research has also found that OA has a positive effect on firm performance [32]. Which brings us to our third hypothesis:

- **Hypothesis 3:** *Organizational attractiveness is positively related to employee service quality provision.*

### The indirect effect of OA

In Fig. 1, OA appears to have an indirect effect or a mediating role in the relationship between internal market-oriented culture (IMOC) and the three response variables. Figure 1 also suggests that IMOC is positively associated with OA. Each of these linkages is discussed in the following.

Figure 1 relates IMOC to OA. The culture is an essential aspect of most organizations. This is no less true of healthcare organizations, where it figures into the competitive advantage they aspire to, being as they face recurring turnover issues [33]. Consequently, we think there are good reasons to include organizational culture in this study and to explore its linkage to employee perception of OA, in addition to the three response variables (Fig. 1). To our knowledge, no previous research in healthcare has examined these linkages from the perspective of current employees.

Schein suggested that “organizational culture is the pattern of shared basic assumptions” [34]. In our study, we view it from a frontline perspective, which is essentially that of the nursing staff, whose basic function can be thought of, in economic or market terms, as “manufacturer of services”—i.e., offering healthcare services to patients. So here we refer to this type of organizational culture as an “internal market-oriented culture,” or IMOC. The concept, not surprisingly, has its origins in marketing [35], which helps explain why its principle notion is to consider employees as yet another form of market—specifically, a market of internal customers who must be handled, or “served,” in the most satisfactory way. Given this perspective, it is crucial for managers to be doubly oriented—toward both the internal market (of employees) and the external market (of customers). Actually, it’s most critical that managers focus *first* on caring for, and treating well, their internal market of frontline employees, because these people are *then* responsible for delivering services to the external market or the organization’s customers [36]. Inevitably, there is a spillover effect, or transference, between managers’ treatment of the internal market (employees) and employees’ treatment of the external market (customers).

Leekha Chhabra and Sharma state it this way: it is “commonly accepted that internal characteristics are transferred to the external environment via the employees of the organization” [36, p, 49]. Thus, once they learn to regard their employees as a form of (internal) market, managers will naturally be more likely to both ascertain their needs and wants and then to actively honor them. The concept of IMOC, as a type of organizational culture, captures frontline employees’ experience, beliefs, and expectations regarding the degree to which managers actually care about them. Thus, IMOC encompasses the more tangible or visible aspects of organizational culture—that is, the observable norm-based behavior that constitutes organizational culture [37].

IMOC is made up of three systems: (1) internal-market intelligence generation, (2) internal intelligence dissemination, and (3) response to internal intelligence. These three systems are closely related and imply a logical flow of information (or intelligence) from system #1 to system #3. Internal-market intelligence generation concerns managerial activities related to collecting information about employees’ needs and wants. It will involve communication between managers and employees as well as that between managers of different departments in the organization. The object of this communication is to develop a common, and granular, understanding about employees’ actual desires. Response to internal intelligence concerns the initiation of concrete managerial behavioral actions based on what they’ve learned.

Previous research has found that employee perception of organizational culture relates to both employee attitudes and behavior [38, 39]. O’Reilly and Chatman argue that culture is indeed about defining attitudes and behaviors [40]. Leekha Chhabra and Sharma found that “the most preferred organizational attributes are organizational culture” ([41], p. 53). It is therefore reasonable to assume that frontline employees’ experience with, and expectations of, an organization’s IMOC is significantly related to their attitudes about its attractiveness. In other words, IMOC is related to whether an organization is *a great place to work*. Similar to external customers who’ve enjoyed good experience with a specific firm brand, it is possible to imagine that internal customers (frontline employees) have good experience with the organizational brand manifested in the concept of IMOC. Consequently, employees’ perception of IMOC represents the internal “living the brand” that employees actually experience in an organization. The beauty of IMOC lies in the eye of the beholder—in this case, the frontline employees. Which brings us to our fourth hypothesis:

- **Hypothesis 4:** *Internal-market oriented culture is positively related to organizational attractiveness.*

In Fig. 1, OA is suggested to play a mediating role between IMOC and the three response variables, which means that each of these variables (TI, EG, and SQP) can be an effect of OA, given employees’ experience of the IMOC in the organization. Previous healthcare research that has focused on frontline employees such as nurses argues that “the culture of a health care organization can be a powerful attribute” ([23], p. 20). Managers play a key role in fostering an organizational culture. We can therefore reasonably assume that managers’ ability to foster IMOC in the organization—manifested in their ability to recognize frontline employees’ needs and wants and respond to them—will positively contribute to employees’ perception of OA. Furthermore, if employees find their managers successful in building an IMOC, and thus perceive their organization as attractive, it will subsequently color their attitudes about TI, as well as their general engagement in their work role and willingness to go the extra mile to provide top service to hospital patients. When the opposite is the case—employees feeling taken for granted, or worse—it will simultaneously erode or even destroy OA and cause a significant negative impact on employee TI, EG, and SQP. There is an implicit assertion here that OA indeed plays a central role in this relationship, as visualized in Fig. 1. Previous research in the field supports the view that OA is of major importance [4]. Although it hasn’t been tested in previous research, there are plausible reasons to assume that OA plays a key (mediating) role between IMOC and our three response variables TI, EG, and SQP.

Our fifth hypothesis is divided into three separate parts:

- **Hypothesis 5a:** *The relationship between internal market-oriented culture and employee turnover intentions is mediated by organizational attractiveness.*
- **Hypothesis 5b:** *The relationship between internal market-oriented culture and employee engagement is mediated by organizational attractiveness.*
- **Hypothesis 5c:** *The relationship between internal market-oriented culture and service quality provision is mediated by organizational attractiveness.*

## Methods

We conducted our study in public hospitals located in southeast Norway. The directors of six of them were contacted, fully informed about our aims, and then invited to participate. Four of the directors agreed to take part. A total of 1104 questionnaires were then distributed by e-mail to their staff, which also included details of the aims and overall focus of the study. We informed the invitees that their participation was strictly voluntary, and that all responses would be handled confidentially. Further, we provided an estimated time to complete the questionnaire. Approval by the Norwegian Social Science Data Services to collect data was also given. Invitees were informed of the name and telephone number of a researcher to contact if they had any questions or comments regarding the study. Checkbox software was used to collect the data. In total, 164 questionnaires were returned, and these were used as the basis for statistical analysis and to test our hypotheses. Although several invitations were sent out, we only achieved a response rate of about 15% (14.85%). There are at least two potential reasons for this relatively low response rate. First, when data for this study were collected the IT platform of the hospitals had changed. Some participants reported being unable to open the link to the questionnaire in places with the old IT platform. Second, those invited to participate in this study were nurses. Given the nature of their jobs (which does not typically include working with computers), it could be that some potential participants did not check their e-mail during the data collection period. Although our inspection of the characteristics of the data shown in Table 1 reveals no obvious or specific selection biases. The data and findings should be interpreted in light of the low response rate in this study.

**Table 1:** Personal characteristics of the study sample (*N* = 164)

		%
Sex	Female	93.3
	Male	6.7
Work as:	Nurse	43.9
	Specialist nurse	49.4
	Midwife	6.7
Employed:	less than 5 years	20.7
	between 6 and 10 years	15.3
	more than 10 years	64.0
Part-time or full-time:	part-time job	50.6
	full-time job	49.4
Age:	younger than 40 years	34.8
	between 41 and 50 years	29.9
	older than 50 years	35.3

Table 1 provide some personal characteristics of the sample. Some 93% of the employees were woman. The reasons for the high number of female nurses are rooted in contextual conditions in Norway where 9 out of 10 nurses are female nurses [42]. Consequently, the responses achieved is reflecting the actual population of nurses in Norway. Most invited employees worked as nurses or specialist nurses. The employees had considerable experience: 64% had worked in the investigated hospitals more than 10 years. About half of the employees worked full-time. Some 35% were under 40 years of age, about 30% were between 41 and 50, and about 35% were older than 50.

### Instruments

The items for the constructs included in this paper is a part of a larger questionnaire published in the master thesis of Lupina and Gravingen [43]. The authors of this paper were also involved in the entire process of developing the questionnaire and in the data-gathering process. All items for each individual construct originated from previous research but were adapted to this specific study. To ensure the best items for each construct, we held several workshops that involved both experts from academia and employees from the target group. During that process, several changes were made with respect to the content of how each construct was defined and to tailor the questionnaire to frontline employees in a hospital healthcare context. The items included for each construct are listed in Table 2.

**Table 2:** Results of the measurement model for the constructs’ internal market-oriented culture, organizational attractiveness, turnover intentions, employee engagement, and service quality provision

Construct	Question items	Loading > 0.4	RRC > 0.7	AVE > 0.5
*Internal market-oriented culture (IMOC)*			0.962	0.679
	Employees have the opportunity to discuss their needs with management.	0.832*		
	Training is seen in the context of individual needs.	0.739*		
	The management is being encouraged to meet to discuss issues concerning their employees.	0.821*		
	I believe management will spend time talking to me when I need it.	0.765*		
	Management understands the needs of employees.	0.899*		
	Management wants employees to enjoy their work.	0.861*		
	I believe that management shows a sincere interest in any problems I have doing my job.	0.896*		
	I believe that management understands that personal problems may affect my performance.	0.779*		
	The division’s policies help meet employees’ individual needs.	0.854*		
	Management meets regularly to discuss issues related to employees’ challenges.	0.822*		
	If an employee from my department is faced with a serious problem, the managers in my division are notified immediately.	0.682*		
	Management works hard to accommodate employees’ needs.	0.906*		
*Organizational attractiveness (OA)*			0.868	0.762
	If a good friend of mine were interested in a job like mine in this organization, I would strongly recommend it.	0.851*		
	If I had to decide all over again whether to take a job in this organization, I would.	0.894*		
*Turnover intentions (TI)*			0.874	0.697
	I often think about resigning from my job.	0.843*		
	It would not take much to make me resign from my job.	0.824*		
	I will probably be looking for another job soon.	0.836*		
*Employee engagement (EG)*			0.852	0.672
	I am so into my job that I lose track of time.	0.722*		
	This job is all-consuming; I am totally into it.	0.945*		
	I put my soul into my job.	0.776*		
*Service quality provision (SQP)*			0.908	0.726
	In my view, I offer good patient service.	0.923*		
	In my view, I offer patient services of very high quality.	0.886*		
	In my view, I offer the patients a high degree of service.	0.887*		
	Generally, I deliver superior service in every way.	0.692*		

* *p* < 0.05. RRC Raykov’s reliability coefficient. ,AVE Average variance extracted

This study focuses on the concept of OA, which measures people’s attitude toward the organization for which they work. The items used for this construct were based on Trybou et al. [4] and were adjusted for our study context. The items used for IMOC were based on Gounaris [44] and were likewise modified. The items used for employee TI were based on Boshoff and Allen [45] and those for SQP on Slåtten [46]. Finally, items capturing the concept of EG were modified from Anaza and Rutherford [47]. Items for all constructs were measured using a Likert scale from (1) strongly disagree to (7) strongly agree.

### Data analysis

Structural equation modeling (SEM) was used to examine the hypotheses. Estimation of our SEM model proceeded in two steps: first, we established the measurement model (essentially a standard confirmatory factor analysis); next, we tested the structural model [48]. SEM was implemented using the “sem” package in Stata version 15.

For the measurement model, reflective latent constructs were estimated. The measurement model was assessed by examining the following (the rules of thumb below are based on Mehmetoglu and Jakobsen [48]):

- The model goodness-of-fit indices, to check for acceptable fit of the measurement model prior to examining the model’s validity and reliability. In this study, we look at the following model goodness-of-fit indices: the standardized root mean square residual (SRMR), with suggested rule of thumb < 0.1; root mean square error of approximation (RMSEA), also < 0.1; and the comparative fit index (CFI) and Tucker–Lewis index (TLI), both recommended to be > 0.9.
- Indicator reliability, measuring the question item’s loading on the latent constructs, with loadings suggested to be ≥0.4 and statistically significant.
- Latent construct reliability, referring to the proportion of the total variation in the construct’s question items that is attributed to the score of the latent constructs. We compute and report Raykov’s [49] reliability coefficient (RRC) (which should be > 0.7), a measure seen more accurate than Cronbach’s alpha [48].
- Convergent validity, to check the extent to which a set of question items reflecting the same latent constructs is positively correlated. The average variance extracted (AVE) should be > 0.5.
- Discriminant validity, which tests the degree to which a construct is distinct from other constructs. All AVE values should be larger than the squared correlations among the latent constructs.

Convergent and discriminant validity are in this setting two subtypes of validity that make up construct validity. When a sound measurement model is established, the structural model can be assessed. The first step is to examine the model fit, using the same fit measures and rules of thumb as for the measurement model. Assuming these are satisfactory, we can examine and interpret the structural model’s path coefficients, similar to examining the parameters in a linear regression analysis. Using standardized values, they range between − 1 and 1. The closer a path coefficient is to ±1, the stronger is the relationship. Since the hypotheses tested in this study are one-sided, the statistical tests are also based on one-sided tests.

To take potential observed heterogeneity in personal characteristics into account, multi-group tests of the structural model’s path coefficients were included [50]. We did two-group analyses, with the loadings constrained to be equal across groups, ensuring that the latent variables have the same meaning in both groups.

Testing of mediation hypotheses was conducted using the “medsem” package in Stata [51], which uses the approach proposed by Zhao et al. [52]. It estimates, within the SEM framework, all possible regression models simultaneously, including latent constructs, and then tests all possible relationships.

## Results

### Measurement model

The measurement model/confirmatory factor analysis had RMSEA of 0.059 and SRMR of 0.045, with both measures below the recommended level of 0.1. Both the CFI and the TLI were above the commonly accepted level of 0.9 (CFI = 0.958; TLI = 0.953). Thus, we conclude that the fit of our measurement model is satisfactory. In Table 2, all of the loadings of the question items were clearly above the threshold of 0.4, lending support to our model. Furthermore, measurement of latent construct reliability using RRC indicated that all five latent constructs were > 0.7, the minimum level of reliability. AVE values are all above 0.5 (Table 1) and thus indicate no problem with convergent validity. Finally, AVE values were larger for all constructs than the squared correlation between them, indicating no issues with discriminant validity.

### Structural model

**Fig. 2:**
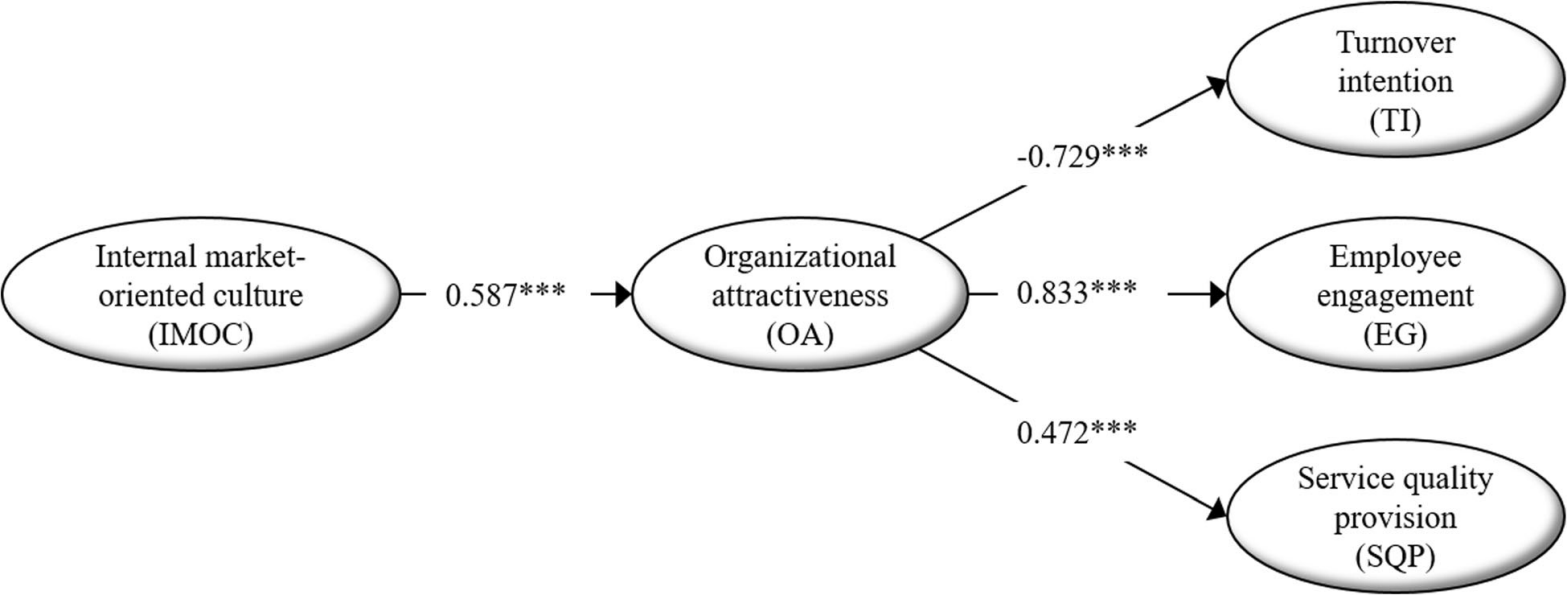
Results of the structural model to analyze frontline employees’ perception of organizational attractiveness. Standardized coefficients (*** *p* < 0.01)

The goodness-of-fit indices were within the commonly accepted thresholds, indicating a sufficient fit of the structural model (RMSEA = 0.062; SRMR = 0.061; CFI = 0.958; TLI = 0.953). Hence, the structural model was examined further. Figure 2 presents the structural model including standardized coefficients for the estimated parameters and their significance level.

We find that IMOC has a statistically significantly positive effect on OA. The results show a statistically significant direct effect between OA and the response variables represented by TI (negative effect, β = − 0.729), EG (positive effect, β = 0.833), and SQP (positive effect, β = 0.472).

Potential observed heterogeneity in personal characteristics and its effect on the structural model are tested with multi-group comparisons, and results are reported (see Additional file 1). We investigated differences in the structural model’s path coefficients for the following characteristics: age, part-time vs. full-time job, and experience. While there are some differences in the direct effects depending on these personal characteristics, the sign and size of the path coefficients show mainly the same results as the basic model, and imply robustness in our findings.

Table 3 presents the results for the mediation analysis. The main associations between the latent constructs in our basic structural model (Fig. 2) are almost identical in our model for mediation analysis. Although there was no significant direct effect between IMOC and EG (β = 0.014), we found a significant indirect effect (β = 0.468), which can then be interpreted as an indirect-only (full) mediation effect of OA. The same result applies for SQP. We found no significant direct effect between IMOC and SQP (β = − 0.020), but a significant indirect effect (β = 0.276), and again an indirect-only (full) mediation effect of OA. There is a statistically significant negative direct effect between IMOC and TI (β = − 0.199), and the indirect effect is significant (β = − 0.342). However, since the direct effect between IMOC and TI is not highly significant, the test by Zhao et al. [52] indicates complementary mediation—that is, OA has a partial mediation effect on the relationship between IMOC and TI.

**Table 3:** Standardized direct, indirect, and total effects of internal market-oriented culture and organizational attractiveness

Effect			Direct	Indirect	Total
			β	β	β
IMOC	➔	OA	0.567^**^		
IMOC	➔	TI	−0.199^**^	−0.342^**^	− 0.540
IMOC	➔	EG	0.014	0.468^**^	0.483
IMOC	➔	SQP	−0.020	0.276^**^	0.256
OA	➔	TI	−0.602^**^		
OA	➔	EG	0.826^**^		
OA	➔	SQP	0.487^**^		

Notes: IMOC Internal market-oriented culture, OA Organizational attractiveness, TI Turnover intentions, EG Employee engagement, SQP Service quality provision. * p < 0.05, ** p < 0.01

## Discussion

Our purpose with this study was to explore the role of frontline employees’ perception of OA in hospitals. Trybou et al. note regarding OA: “although the concept [OA] … has received a lot of theoretical attention, relatively few empirical studies have examined this issue” ([4], p. 2). Previous research on OA has limited its scope to mostly focus on attracting potential applicants to the organization [11, 12]. In contrast, our own study focuses on current employees’ (specifically frontline employees’) perception of OA.

This study makes four contributions. First, it examines the concept of OA from the perspective of current employees. To our knowledge, this is only the second study within healthcare research that takes such a perspective. Second, it links OA to important job-related outcomes of hospitals, specifically referring to turnover intentions (TI), employee engagement (EG), and service quality provision (SQP). No previous study within healthcare research has examined the association between these outcomes and employees’ perception of OA. As such, it is a direct response to the suggestions of Trybou et al. [4] that future research should also examine potential outcomes of OA. Third, it examines how a frontline-related culture concept—an internal market-oriented culture (IMOC)—acts as a determinant of OA. By including IMOC as antecedent to OA, our study responds to Trybou et al.’s admission: “we do not yet know what determines attractiveness for those people already working at the organization” ([4], p. 2). Fourth, by simultaneously studying both IMOC as determinant or stimulus to OA, as well as the responses of OA (referring to TI, EG and SQP in Fig. 1), we elucidate the role of OA from the perspective of frontline employees. No previous research has examined these variables in relationship to hospitals’ frontline employees’ perception of OA, thus making this study a unique contribution to health-service research.

The core variable of this study is OA. Here, it embraces frontline employees’ deeply held attitudes [4] regarding whether they would (1) work for the same organization again if given the choice, and (2) recommend the organization to a close friend. Frontline employees in healthcare organizations work in knowledge-intensive institutions [53], sometimes referred to as “professional service firms” [54]. The word “frontline” implies that these employees play a key role in the organization. Zeithaml et al. suggest that this role is not primarily limited to “only” providing services but that these employees represent (1) the organization in the eyes of customers, (2) the brand, and (3) the marketers of the organization [55]. Consequently, the effects of this role clearly go beyond just providing services. In this study, three effects of OA are examined (referring to TI, EG, and SQP in Fig. 1). The findings reveal empirical support that OA is linked to all three responses. However, when comparing the strength of each individual responses or effect, OA was shown to have the most impact on employee engagement (EG), followed by turnover intentions (TI), and finally service quality provision (SQP).

The findings reveal that OA has a direct effect of EG (β = 0.833). In total, OA explained 69.5% of the variance in EG, which can be described as substantial explanatory power. The concept of EG has been defined as “a positive, fulfilling, work-related state of mind” ([25], p. 74). Clearly, frontline employees’ attitude toward the OA is a substantial driver for EG. In previous research, EG has been emphasized as an important domain to focus on [27, 28]. For example, recently Slåtten and Lien stated regarding EG: “there is need for more extensive research into several new areas and aspects related to this interesting and seemingly important construct” ([28], p. 97). Our study contributes new knowledge by broadening the scope of previous engagement research, revealing that OA is able to promote frontline employees’ EG. This highlights the importance for hospital managers to regularly collect information from frontline employees in their organization on their attitudes about the attractiveness of their organization. Identifying the level of attractiveness that exists in an organization and responding to such information in a positive, appropriate manner contribute to fully engaging frontline employees and capturing “their minds and hearts at each stage of their work lives” [29]. Thus, following Werner, OA can be considered a type of organizational *resource* or *asset* [56] because it can be characterized as a valuable, inimitable, and nonsubstitutable resource or asset for the organization. Consequently, based on the criticality or key role of OA for EG, hospital managers should strive to capitalize on this organizational resource or asset, as they have the potential to substantially strengthen frontline EG in their organization.

Although OA was identified as the most significant driver to EG, the findings reveal that OA also has a substantial direct effect on employee TI (β = − 0.729). OA explains 53.2% of the variance in employee TI, which is considerable. This finding is an interesting one due to the high turnover rates among nurses worldwide [57]. Chen et al. emphasize that “it is of importance and urgency for hospitals to retain excellent nursing staff” ([30], p. 1). The strong association between OA and TI imply that OA is a key antecedent to lower frontline employees’ TI in hospital organizations. Considering all the costs incurred when employees leave an organization—costs for recruiting, training, motivating, and onboarding new employees—lowering employee TI contributes significantly to the hospital’s bottom line. One reason why OA has such a substantial impact on frontline employees’ TI can be found in psychological contract theory. Trybou et al. note that “psychological contract theory is considered to be one of the most influential theories to understand organizational behavior” ([4], p. 2). As the name “psychological contract” suggests, this theory, rather than focusing on the written contract binding an employee to an organization, focuses on the intangible contract—we might call it a “mind-set contract”—that psychologically binds that employee to an organization. Specifically, the term “psychological contract” refers to “the way the working relationship is interpreted, understood and enacted” ([4], p. 2). Thus, the basic notion of how OA is defined in this study has much in common with core aspects in the definition of “psychological contract.” Both concepts focus on a person’s beliefs and attitudes. So it is reasonable to assume that OA implicitly includes a psychological-contract element that potentially binds the employee to his or her organization. Depending on the strength and direction of OA (“direction” referring to whether the attitude is positive or negative), this furthermore is related to the two main responses of an employee, namely, *Should I stay or should I go?* As highlighted by the findings from this study, OA has strong explanatory power on TI. Clearly, hospital managers should strive to capitalize on OA as it has a significant ability to lower TI of frontline employees.

These employees play a key role in a hospital, for they physically represent it when providing services to patients. During what has been called “the moment of truth”—when frontline employees interact with patients—“the willingness of employees to engage in discretionary effort determines the level of SQ [service quality] delivered to customers, leading to customer satisfaction” ([58], p. 2594). In our study, “service quality” refers to the perception of frontline employees as to the quality of the “design and delivery of knowledge-intensive solutions” ([31], p. 1603) to hospital patients. The findings reveal that employees’ attitude regarding the attractiveness of their organization (OA) drives frontline employee SQP (β = 0.472). The linkage between OA and SQP has not been examined in previous health-service research. But this linkage is supported on the basis of Heskett et al.’s service-profit chain model [59]. A central premise of this model is that internal factors of a service organization affect factors external to it. Based on the service-profit chain model, one such internal aspect would be how frontline employees perceive OA of their employer. Our findings reveal that OA has a direct effect on the performance level of SQP to hospital patients. This finding is supported in previous research showing that OA has an impact on firms’ performance [32]. A practical implication of this is the importance for hospital managers to actively “control” the level of frontline employees’ perception of OA, and to take whatever steps are needed to develop, maintain, or increase OA to ensure that it keeps within a (positive) zone of tolerance.

In this study, “IMOC” refers to norm-based behavior and thus concerns the most observable parts of organizational culture [37, 60] directed toward frontline employees. The findings reveal that IMOC is closely related to frontline employees’ perception of OA (β = 0.587), explaining 35% of the variance in OA. With reference to nurses working in healthcare organizations, Park and Kim note that culture “can be a powerful attribute” ([23], p. 20). The findings reveal that the relationship between IMOC and EG and SQP is fully mediated of OA. Moreover, there is a (statistically significant) direct linkage between IMOC and TI (β = − 0.199), as well as a (statistically significant) indirect linkage between IMOC (via OA) and employee TI (β = − 0.342). It clearly highlights how IMOC is a triggering or *Stimulus* factor for OA as well as TI, EG, and SQP. This finding supports previous work that stresses the importance for managers to orchestrate the appropriate culture in an organization. The culture in an organization is something that is emerging and dynamic [61]. It is the “first step toward creating satisfactory work environments” ([62], p. 462). Kucherov and Zavyalova classify organizational culture as a psychological attribute of an organization’s employer brand ([63], p. 90). Accordingly, hospital managers should do whatever is necessary to nurture IMOC and should themselves be good role models in their daily managerial practices by actively living the employer brand with respect to IMOC in the organization. On the basis of this study, hospital managers must recognize that IMOC and OA are fundamental attributes to accomplish high-order objectives of the organization such as employee engagement (EG), delivery of excellent service quality (SQP), and reduced turnover intentions (TI) among current frontline employees.

### Limitations and future research

The approach adopted here of focusing on OA from an internal frontline employee perspective has been lacking in health-service research. It holds the promise of a number of opportunities for future study. We might cite four in particular.

First, because our study is limited to just one type of frontline hospital employee, namely nurses, our findings might not be generalizable to other groups of frontline employees in the same context.

What about, say, doctors? Previous research has indicated some differences between how they and nurses perceive their organization. One identified difference is in job satisfaction [64]. Future research could identify whether OA plays different roles with other frontline employees and whether the antecedents and effects of OA are significantly different compared with our findings.

Second, because this study explored just three effects of OA, future studies might explore a broader view of effects. One example is the relatively new concept of organizational readiness for change [65]. Because change is inevitable in any organization, it is important to understand *why* and *how* frontline employees engage in behaviors associated with change. One approach is to investigate whether frontline employees’ perceptions of OA can be characterized as enabling or promoting factors regarding organizational readiness for change. Other direct effects of OA worth examining include employee commitment, employee productivity, innovative behavior, and dimensions of learning on an individual level, team level (relationship learning in teams), as well as learning on a more organizational level. Including a wide range of effects would provide more detailed knowledge on the role and actual value of focusing on OA in hospital organizations.

Third, although our study found IMOC to be associated with OA, future research could also include other factors that potentially promote OA. What, for example, is the effect of different leadership styles, such as transformational leadership, transactional leadership authentic leadership, empowering leadership, and ethical leadership? Or how about studying leadership from a role-model or social-learning-theory perspective? Because of their power position, leaders are strong norm-senders with respect to their culture. Specifically, based on social-learning theory, future research might examine whether a supportive and cooperative leadership style promotes OA as well as a supportive, cooperative climate in the organization in general. Simultaneously, it might examine how these concepts are linked to OA and other potential outcomes. Such a focus would deepen our insight into several critical aspects related to OA and thus contribute both theoretically as well as practically in improving managerial practices.

Fourth, the concept of IMOC was chosen because it is especially pertinent to frontline employees in hospital organizations. Our findings reveal that IMOC is a substantial variable that fosters OA. Although the inclusion of IMOC clearly contributes to our understanding of what causes OA, future research might include other types of organizational culture as well as types of national culture that are well accepted in the literature. With respect to organizational culture, for example, types of culture from the so-called competing-values framework could be included [66]. This framework covers four types of organizational culture: (1) market, (2) adhocracy, (3) clan, and (4) hierarchy [66]. Moreover, for national culture, future research might include one or more dimensions of national culture as suggested by Hofstede [67], who identifies four dimensions of national culture: (1) individualism, (2) power distance, (3) uncertainty avoidance, and (4) masculinity [67]. One might also explore whether the suggested types of culture, either individually or in combination, can have direct or indirect impacts on employees’ perception of OA. Including these aspects of organizational and national culture would contribute significantly to our understanding of what drives OA among frontline employees. Identifying the “right culture” will reveal the critical first step that has the potential to create the desirable responses (e.g., excellent service quality, lower turnover intentions, etc.) and thus contribute to hospitals achieving a competitive advantage.

## Conclusions

This study examines the role of OA from a frontline perspective in hospital organizations, an area of study previously overlooked. It reveals the value for hospital managers to develop an internal market-oriented culture (IMOC) directed toward hospital frontline employees, as it has both a direct effect on OA and an indirect effect on frontline employees’ engagement, turnover intentions, and service-quality provision. Consequently, OA plays a key role, reflecting or signaling frontline employees’ perception of whether their organization is indeed a great place to work.

As for hospital managers, it is now demonstrably important for them to consider OA as a core organizational resource or asset—something that needs to be focused on, maintained, and cultivated if they are serious about making their workplace highly attractive in a very competitive market.

## Additional file

Additional file 1: Multi-group comparisons. This file contains multi-group comparisons of age, part-time vs. full-time job and experience (DOCX 141 kb)

## Abbreviations

AVE: Average variance extracted
CFI: Comparative fit index
EG: Employee engagement
IMOC: Internal market-oriented culture
*NSD*: *Norwegian Social Science Data Services*
OA: Organizational attractiveness
OECD: *Organization for Economic Co-operation and Development*
RCC: Raykov’s reliability coefficient
RMSEA: Root mean square error of approximation
SEM: Structural equation modeling
SOR: Stimulus-Organism-Response model TI Turnover intentions
SQ: Service quality
SQP: Service-quality provision
SRMR: Standardized root mean square residual
TLI: Tucker-Lewis index
