# Supplementary material for: The role of organizational attractiveness in an internal market-oriented culture (IMOC): a study of hospital frontline employees
Source: BMC Health Serv Res. 2019 May 14;19:307. doi: 10.1186/s12913-019-4144-8 (PMC6518731; doi:10.1186/s12913-019-4144-8)
Supplement: Supplementary file 1 — Multi-group comparisons. This file contains multi-group comparisons of age, part-time vs. full-time job and experience (DOCX 141 kb) [file 12913_2019_4144_MOESM1_ESM.docx]

Multi-group comparisons of age, part-time vs. full-time job and experience, * indicate significant difference (* p<0.01) across groups for that parameter.
